# The Etiology of Dental Decay

**Published:** 1883-02

**Authors:** Geo. Watt


### ARTICLE II.

### *The Ætiology of Dental Decay.*—(Continued.)

BY GEO. WATT, M. D., D. D. S.,

[Read before the Ohio State Dental Society.]

Because the specific acids, in producing caries are always nascent, as rapidly as atoms of any of them are formed or liberated, they take hold of the tooth substance, when generated or liberated in contact with it. And this renders it impracticable to find the specific exciting agent in any case of dental caries in an uncombined state. The camal was not seen; but the Arab knew it had passed his tent by the fresh tracks it had left behind it. The specific acids —the exciting causes of caries—are not seen, but their work and its results are plainly observable. When this doctrine is clearly in the mind, no one will be surprised to find the buccal fluids alkaline in mouths affected with rapid decay. In a majority of such cases the alkalinity is due to ammonia, which is a compound of nitrogen and hydrogen, and which is always formed during the putrefaction of azotized bodies, or whenever the vitality of such tissues is so low that it is not able to resist chemical action. And when ammonia is exposed to the action of oxygen, either nascent or quiescent, its nitrogen is burned or oxidized into nitric acid, and its hydrogen into water. The great affinity of the acid and the water for each other, Liebig calls a disposing circumstance, which he says increases the tendency to the oxidation. I am not unmindful that the paper I read before you last year was pronounced "partly antiquated," *partly* because it was according to Liebig; but a chemical fact in the days of Liebig is a chemical fact still, in despite of the opinions of juvenile chemistry. Those who are duly posted on the chemical theory of decay, and are, at the same time, wide awake, whenever they find a decidedly alkaline reaction of the buccal fluids, resort at once to a tonic and acidfying course of treatment, as they know the great danger that white decay is likely to run a rapid career, unless the tendency is arrested. And their antiseptic treatment is not used by them for the destruction of germs, but to get rid of the tendency to the formation of nitric acid.

In the discussion of Prof. Spalding's paper in the society, Dr. Swain is represented as saying, "Twice during the year I have ha l in my office a harmlessly insane young lady. In her mouth I have found the saliva uniformly acid, but her teeth do not decay." What acid gave the uniform reaction we are not told; but it was probably the normal carbonic acid, and it was fortunate for her that ammonia was not found to neutralize it. It is unfortunate that such half-way observations and tests tell us so little; but when we think how busy and how tired the ordinary dentist is, we cannot be surprised.

But suppose the saliva, the mixed buccal fluid, is all the time acid, and that the acid or acids it contains are sufficiently concentrated to act on the teeth, nothing like the phenomena of any variety of dental caries can be produced. If the teeth are of uniformly good texture as to their entire external surfaces, these surfaces may be roughened, or corroded by the acids. Further on I shall endeavor to explain how the phenomena of caries are produced, in accordance with the so-called chemical theory, and I think no one will regard the results as at all mysterious.

A paper that has attracted some attention on both sides of the water was read before Section XII of the International Medical Congress, August 6, 1881, by Arthur S. Underwood, M. R. C. S., L. D. S., and W. J. Miles, L. R. C. P. Lond., F. R. C. S. and C.,

I regret the necessity to differ with such careful and such distinguished observers; but truth is truth, and men who, from their learning, have a right to exhaust the alphabet in the display of titles, may be mistaken on very small matters. Their paper has been variously published, but I am using it as it appears in the Journal of the British Dental

Association, where I first noticed it. It seems to require only moderate attention to see that these brethern have not clear ideas as to the "chemical theory" of decay. For example, they say, "With regard to the purely chemical theory, we cannot accept it as wholly satisfactory for the following reasons." Now I doubt if any educated dentist accepts the *purely chemical* theory, ignoring organic or physiological conditions as predisposing causes.

But a consideration of their reasons for non-acceptance shows a decided lack in their understanding of the so-called acid theory. They say: "1. Because the destruction of dentine, effected by the action of acids alone, under a septic condition, does not resemble caries, either in color or in consistency, it being colorless and gelatinous, the process uniformly attacking all parts of the surface.

They refer to the useless experiment which our boys used to call "tooth pickling." Putting dentine to soak in, say hydrochloric acid, the lime salts will be dissolved, and the gelatin will be left. But the acid is quiescent, and not, therefore, in the condition necessary for the imitation of caries. They are enveloped by the same cloud which overshadowed Prof. Spalding. In their third reason they gravely tell us: "Further, our own flasks show that maliac and butyric acids with saliva in a meat infusion, have not, under aseptic conditions, produced caries." Most certainly not; for these acids never did, and never will produce dental caries under any conditions. Chemical action is definite; and where but a few reactions are observable, as is the case in caries, but few, and just as few, reagents are concerned in the production, and butyric and malic acids are not two of them. They say, too, in this connection, "that acids alone do not alone destroy a living tissue;" and they apparently try no enforce the statement by the remark, "that the stomach is not digested by its own acids until it has been removed from the body." But they surely do not mean to include sulphuric, nitric, hydroflouric acids, and the like, in their statement that "acids alone do not destroy a living tissue."

I will quote the fourth reason in full, and think it is plain that their inferences are not legitimately drawn, while the closing remarks show that they have no true idea of the position held by the advocates of the acid theory. They say : "4. Lastly, we would urge that, when caries occurs in the mouth, it is always under circumstances more favorable to the action of the germs than to that of acids. There is always, first of all, a minute pit or haven where germs can rest undisturbed and attack the tissue. We cannot, upon the purely acid hypothesis, explain why the same acids that originally caused the decay, gaining access through some minute imperfection of the armor of the enamel, do not in the same mouth, or under the same condition, attack the wounded enamel at the edges of the filling. The germs cannot rest there, they are constantly washed away if the surface is fairly smooth ; but the acids literally bathe the part except during the performance of the act of mastication, when the alkaline parotid and submaxillary saliva neutralizes their action."

Now can any one believe that "a minute pit or haven" is a better place for the development of germs than for the formation of acids ? And the germs are always washed away from the edges of the filling while "the acids literally bathe the part." Why do not the germs "stick in their holders," as the Irishman did when trying to ride the mule ? They need not be washed away if they are like Prof. Mayr's little pets, able to crawl through the holes in normal enamel. (But they do that just to show Prof. M. how small a hole can be crawled into rather than give up a favorite theory). Their allusion to the acids bathing the parts show that they have not caught the first idea in the acid theory of decay. They are bemixed in the slough of quiescent acids, with Prof. S., whom we quoted at the outset. But if a nascent acid is to cause caries, it needs the "minute pit" for its development more than do the germs, It is very difficult to form, either by synthesis or analysis, a nascent acid at the junction of a well-finished filling with the border of the cavity.

But having got thus far, these gentlemen find their progress arrested, unless they come over to the acid theory. Hear them : " Most probably the work of decalcification, is entirely performed by the action of acids, but these acids are, we think, secreted by the germs themselves." " *We think*," is good. But what acids do the germs secrete ? Chemical action is definite, and but three or four kinds of " decalcification " occur in dental caries.

Time and space will not allow a notice of all the points assumed by these brethern. Let me notice an item or two in their summary. They say : " 1. We consider that caries is absolutely dependent upon the presence and prolifications of organisms. That these organisms attack first the organic material, and feeding upon it, create an acid which removes the lime salt." First they beg the question, —at least we may claim the Scotch verdict of " Not proven "—and then they *guess* the little bugs, or buggers, create an acid. Well, what acid ? In the experiments, occupying over ten years, with results reported nearly a a quarter of a century ago, I was not content to guess at the acids. True, in the most common variety of dental caries I did not find hydrochloride acid dissolving the sulphosphate, and decomposing the carbonate of lime, for the acid being nascent, each atom was saturated as fast as formed ; but I did show chloride of calcium pressed out of the softened organic matter remaining in the cavity, and precipitated bone phosphate from solutions obtained from similar cavities. If I didn't exhibit the dragon, I showed his tracks. And instead of the organic matter being first attacked, as those brethern claim, much of it is often found in the cavity, and so little disturbed as to distinctly show its organic structure. And Prof. Mayr, finding the lime salts present in an entirely different form of caries, does not in the least contradict this statement.

Further in their summary they say : " 2. That suppuration of the pulp and its sequelæ, such as alveolar abscess, depend also upon the successful working of organisms."

This claim is now common, and was made, in noticing one of the *Journal's* Specials, perhaps, by Prof. Mayr, he going so far as to claim, if my memory is not treacherous, that no one had ever held that such action could originate except through the action of germs. The position is held by many of my friends, and by distinguished men for whom I have the highest respect; but they are in error nevertheless. This may appear from various considerations, but, first look at the following: The *British Medical Journal* not long ago said this: "A contribution of great importance will be found in the October number of Virchow's *Archiv.* Dr. Uskoff, of Cronstadt, has made experiments which appear to prove that suppurative inflammation can occur independently of micro-organisms. He injected small quantities of distilled water, milk, oil, pus and turpentine into the subcutaneous cellular tissues of dogs, and examined the tissues microscopically a few days later. He found that the injection of bland fluids, such as water and milk, caused no suppuration, provided that only a gramme or two was injected, but that large abscesses formed when several grammes were introduced. The injection of turpentine, especially when not diluted with oil and carbolic acid, produced, in most cases, large abscesses, containing pus, which was entirely free from micro-organisms. From this, Dr. Uskoff, concludes that, although micro-organisms may, in many cases, be the proximate cause of suppuration, morbid process may exist entirely independently of them, being due to chemical irritation, as when turpentine is used, or to damage of tissues, as in cases where a considerable amount of water is forcibly injected. We intend shortly to refer, at greater length, to this important subject."

Few stand higher than does Dr. Uskoff as an exerimenter and close observer. Years ago, without any knowledge of other researches in this direction, the writer very carefully conducted a series of experiments, with direct reference to this subject, which fully confirm the conclusions arrived at now by Dr Uskoff. The record of these experiments, conducted mainly at No. 55 W. Seventh Street, Cincinnati, was destroyed by accident. In many of them I had the counsel and assistance of the late Prof. Thomas Wood, whose name is a guarantee of accuracy.

I had also the cöoperation of the late Prof. W. H. Mussey, who, although quite too busy to give immediate personal attention, gave most valuable suggestions as to the methods of conducting the experiments, especially with reference to securing aseptic conditions of the appliances used. The hypodermic instruments used were rendered aseptic partly by heat, and partly by carbolic acid, etc. Distilled water and other agents, free from micro-organisms, were introduced into both cellular and muscular tissues of human beings, dogs, etc.; and when it was ascertained that suppuration had taken place, aseptic conditions being still observed, with the hypodermic instrument, pus was drawn out and found entirely free from micro-organisms. This is but a flimsy account of the experiments of prof. Wood and myself. And while I have never made a claim to be an expert with the microscope, still I think I could see a beetle or a June-bug, if in full view. But Prof. Wood had few superiors in this line. Had it not been that his native modesty held him back, he would have been recognized according to his merits. It is more than gratifying to find the researches of Dr. Uskoff confirming the teachings of our experiments of 1867 and 1868, which I had repeated, as opportunity served, many times since, and usually, when on human subjects, taking the precaution to steal up the orifice made by the hypodermic point, with collodion. Prof. Wood and his son are both dead, and it is not probable any notes of these experiments can be found among his papers; and I am not sure that he took notes of them. He took none in our nitrous oxide investigations, relying on his very tenacious memory.

If I had not implicit confidence in the testimony of these experiments, they would not be here referred to, and though it is a delicate subject, you will allow me to allude to the fact that my physical disability has given me ample leisure to experiment, and a few of you know that the time has been improved.

A carefully prepared paper was read on "The Etiology and Pathology of Dental Diseases," at the annual general meeting of the Association at Liverpool, by Henry Sewill, M. R. C. S., L. D. S., England. In reference to caries he says: "Caries must be defined as a process of disintegration, commencing invariably at the surface, and proceeding inwards, affecting dentine more rapidly than enamel, and due entirely to external agencies. We are now positively assured of the truth of these facts; that caries is not an inflammatory change, that it does not depend upon any connection, vascular or nervous, with the rest of the body, and that caries may occur even in an extracted tooth, which is retained in the mouth by artificial means, as on a denture or pivot."

Further along he says: "The active agents in caries are, acids and living organisms." The acids, maliac, buytric, and acetic, are the products of chemical change and fermentation set up in fragments of organic matter, food, mucus, and epithelial scales, which are commonly present in the mouth."

A casual remark often shows an opinion or an error as well as a labored essay could. Mr. Sewell has this remark: "That the acids alone do not produce all the phenomena is obvious from the fact that dentine, after perforation of the enamel, is the favorite seat of caries; whereas acid acting alone would most rapidly attack the enamel. It is equally inconceivable that micro-organisms could gain access to the dentine without the assistance of an acid capable of perforating the enamel." A little further on he says: "The initial stage of caries undoubtedly depends greatly, if not entirely, on the action of acid, and this stage, let us note particularly, may be induced artificially by introducing a pellet of cotton wool between two teeth and allowing acid to collect in it and to act upon the adjacent enamel."

In the first quotation here the writer has forgotten that enamel is much harder than dentine, and that cohesive attraction is one of the modifying circumstances of chemical affinity. Marble and chalk are chemically the same, and an acid acting on them yields alike results; yet it acts with much greater energy on the chalk, simply because less cohesion is to be overcome. This quotation, too, shows, that the writer fails to recognize the facts that acids are nascent when they cause caries directly. The experiment with the pellet of cotton wool is instructive; but if he had said "allowing acid to" be *formed* in it rather than to collect in it, he would have been nearer to the correct idea.

The writer is troubled about the color of caries. The black variety is not mysterious. The organic matter is "carbonized," as is a cork sometimes, by sulphuric acid; and animal charcoal is black. And if we take the most common variety of caries, when recent, it is the color of the gelatinous portion of the dentine. This remaining is gradually darkened by oxidation, just as decaying leaves, etc., are darkened from the fact that their carbon is more slowly oxidized than are the other constituents. But I have dwelt too long on this paper, and so it must be abandoned for something later, though some of its other points are worthy of notice.

The last paper it is proposed to notice, in this connection, is from the pen of Dr. C. T. Stockwell, of Springfield Mass., and is entitled "Etiology of Dental Caries. Acids, or Germs; Which?" It was read before the New England and Connecticut Valley Dental Societies, October, 1882, and is, accordingly, up with the times in reference to the principles held by the author.

It is feared that Dr. S. is not well up in the history of professional opinions, for he calls the acid theory, or rather, I suppose he means, the chemical theory, "the old dusty creed," when really it is quite new, never thought of, perhaps, till within the memory of some now living; and the other theory, viz: that dental decay is caries, similar to caries in other bony tissues, has been held ever since the dawn of medical science. Which is old? Which is dusty? Instead of labelling the chemical theory "reopened for further investigation," as he advises, he would better inquire as to the success of the colored brother who tried to resurrect his dead sheep by inflating it with his own breath through a goose quill. The revival is about as likely to occur in the one case as in the other. Not many are likely to believe that dental decay is an ulcer, even though all the force of the *New England Journal* shall continue to eulogize Prof. Mayr, who is trying to revive this almost obsolete idea. Dr. S. tells us that "This conclusion of Prof. Mayr is based upon a study of chemical facts in relation to dentistry covering several years, during which a large number of experiments and chemical tests have been made in which it was found in every case 'that the lime salts are only slightly diminished in the decayed mass compared with the healthy tissue."

Now which variety of decay? Here are experimenters eulogizing each other, and yet so much in the dark that they regard it as of no importance in detailing experiments to give the principal facts. No one expects to find the lime salts removed from black decay, if he has studied the chemical theory; nor does he expect to find the reactions of acetic acid. But Prof. M. seems to think a mare's nest (no play on words) is found because he finds the lime salts, without acetates, in a case of black decay. He did not intend to tell us that it was this variety of caries; but the fact slipped out.

Dr. S. boasts of the "several years" of Prof. M.'s researches in dental chemistry. How many make several? Not very many, as we know; but Prof. M. will not allow that to count, for he insists, if I understand him, that all the long years of Prof. Buckingham's researches amount to nothing. What great discoveries has Prof. M. made in these several years? Great results ought to be heard of, especially since Dr. S. places in his hands nearly every tooth he extracts, and as he extracts sixteen at once, or in a brief period. But again let me remind you, that neither Dr. S. nor Prof. Mayr seem to know that there is more than one variety of decay, though as you all know, the black and white decays are not more alike than a boil and a blister. Only last week a man with heavy gloves on, raised his hands and said to me, "Doctor, my hands are sore. What shall I do for them?" He is from New England, and was definite in describing his hands as are these men in describing the decay of the teeth.

But I asked as to Prof. M.s' great discoveries. Here is one: "The tooth will be destroyed if either the *outer* forces become too strong relatively to the *inner* forces, or if the *inner* forces *become too weak.*" Further down he says, "About the septical theory and its principles there is no longer ignorance among us. We may consider the facts settled. But why does not healthy tissue become attacked like diseased tissue? To explain the fact, we have again to go back to the simpler forms of bioplasson to amœba. Amœba may live in water containing bacteria; but scientifically, the bioplasson of the bacteria becomes a part of the bioplasson of the amœba, the latter being in excess and a more staple compound. The same will happen to a tooth; *bacteria may enter healthy enamel;* their number, from the great density and closeness of the net-work of the enamel, is only small at one time, and the fibrils within, being on guard, so to say, do nothing more or less than dispose of the entering bacteria, by way of *assimilation.* Thus, as long as the fibrils prove stronger, the bacteria will not gain entrance; but let the fibrils become weakened and they will crowd in. The danger of a part of the enamel being taken away lies in the fact that the dentine, being provided with much wider canals, the bacteria may enter in much larger number, and thus overpower the fibrils of the dentine."

I have quoted this at great length. It is either the best specimen of sharp-looking extant, or it shows what small holes things will crawl into for sake of a pet theory. Prof. M. has found a hole to retreat into, smaller than those in the enamel through which the bacteria crept. Even Dr. S., though his eulogist, seems discouraged about following him through the little holes. He says, "The statement of Prof. Mayr, also regarding the possibility of bacteria entering 'healthy enamel's seems, in the light of these investigations, to be less visionary than many have supposed."

Why speak of the *possibility* of the bacteria entering healthy enamel? Prof. M. teaches us they "may," and that there is nothing to hinder, except when the "fibrils" are strong, wide awake and a little hungry, they eat the little buggers as fast as they go in, which is hardly fair play. They ought to let them get fully within, and then stand up in a fair manly fight.

Some have stupidly (?) thought the enamel is a protection to the dentine beneath. It seems the fibrils are the sentinels on picket duty—"being on guard," as Prof. M. puts it. Dr. S. speaks of the entrace to the tubuli being barricaded mineral substances; but what a mistake, when Mayr's bugs crawl right through them. The fibrils are the barricades; and a course that tends to invigorate them and sharpen their teeth, is the proper prophylactic treatment. Nor is it a mistake, as has been intimated, to put in the limesalts, even if acids are the main source of danger; for the teeth were made to grind the food, and fibrils would not answer. Besides, in the structure of the human body, all is made with reference to life Nature has not formed the human frame for death—has made no provision for it.

Near the beginning of this paper I spoke of optical delusions, and that the tendency heretofore increases in proportion to the squares of the powers used. This may explain the many contradictions. They are very abundant with the advocates of the germ theory who claim to tell just what they have seen. We have room to notice but one now: Dr. Miller of Berlin claims to have specimens "bored through and through" by leptothrix, as well as by other micro-organisms; while Dr. Sewill, of England, says the leptothrix "never penetrates below the surface." I am tempted to add that Dr. Sewill says "it is inconceivable that micro-organisms could gain access to the dentine without the assistance of an acid capable of perforating the enamel," while Prof. Mayr sees the little fellows walking right in and playing the mischief, if the fibrils happen to be in delicate health. Seeing the bacteria going right through healthy enamel is the greatest discovery of modern microscopy. Some, who are naturally incredulous, will doubt the statement; but all such will incur the criticism of the *New England Journal*, with its six or eight editors, one of them "scientific." Therefore, be cautious.

Dr. S. seems to think that the chemical theory is squelched because alkaline mouthwashes, etc., fail to serve a good purpose "Acid agents would serve a better purpose," he says. Well, what of it? Can you not bear me witness that in the journals, and before this society, and though an earnest advocate of the chemical theory, I have perseveringly and persistently urged the use of acids as preventitives of decay? Do not Drs. Rehwinkel, Jennings, and others recall my eulogy of saur-kraut for this purpose? How many of you have given me the pet name of "Old Pickle," on account of my almost continually calling your attention in this direction? Was this a symptom of abandoning the chemical theory? Nay, verily! Dr. S., further down, tells us that an alkaline condition of the saliva is as productive of *a certain class* of germ development as an acid condition is of *other* classes." But he seems to care nothing at all whatever for the cause of the alkalinity; but as that condition of the buccal fluids is often, if not generally, caused by ammonia, you will bear in mind that this is a frightful precursor of the most destructive form of dental caries. And in this connection, he urges the importance of correct diagnosis, yet gives it so little attention himself that he seems not to care as to the cause of the alkalinity, nor as to the variety, of caries to be considered.

Dr. S. also quotes from Surgeon George M. Sternberg, of the United State army, "as an indorsement of this position of Prof. Mayr," leaving us to infer that the position to be indorsed is the statement that "bacteria may enter healthy enamel," a position which Dr. S. himself intimates is somewhat "visionary." But the main point in the quotation from the Surgeon is that when vital resistance of tissues is reduced the parasitic organisms can overcome them more readily; but that proves nothing for the germ theory, for as vital power is reduced, chemical affinity acts with greater relative power; for who does not know that vitality is one of the modifiers of chemical force?

Dr. S. tells us that he is "quoting Prof. Tyndall in substance," in saying that if any putrefactible substance is excluded from the air, or if it is exposed to air from which all germinal matter is excluded, no putrefaction will occur. "In other words, no germs, no putrefaction;" but this is flatly contradicted by the experiments of Dr. Uskoff already quoted, as well as by the very careful experiments of the late Prof. Thomas Wood and the writer, as referred to in this article.

But the fearful length of this paper, and nothing else, prevents a reply to every position of Dr. S. adverse to the chemical theory of decay. The remaining ones are more easily answered than are those noticed. One remark of his will yet claim attention and then his paper must be dismissed. He says: "Our attention in the past, has been directed too exclusively to simply the inorganic portions of the teeth. It has been the decalcification of the teeth, the *non-vita limesalts, etc.*, that has (have?) absorbed our attention to the exclusion of a proper conception of the vastly more important part that *protoplasm* plays in the life and existence of these organs,"

Now who has been slighting the organic materials of the teeth? I know of no prominent writer who has advocated the chemical theory of decay who has not stated the action on the organic, as clearly and as fully, as that on the inorganic portions of the tooth, when describing the action of the exciting agent in each of the varieties of caries. In Watt's Chemical Essays, in the articles " Thoughts on Caries," and " Chemistry of the Mouth," I know this will be found true. (See Appendix to Taft's Operative Dentistry.")

With some thoughts on the nature and circumstances of dental decay, this long paper must close.

Many are puzzled to know why caries is found in definite spots, if the chemical theory is the true one. Let it be borne in mind that the acid immediately causing the decay is always nascent when doing its work. Many reasons explain why it is generated, or liberated at particular points. Then the microscope reveals places in both enamel and dentine imperfect on account of imperfect organization. These points yield to chemical action much more readily than normal structure; and this is one reason of the definite position of caries. And now suppose the exciting agent has, through a defective spot, got within the enamel, it acts on the dentine, and is not readily washed away by the fluids of the mouth, or even by the brush. Take, for illustration, a case of white decay. The exciting agent is here able to break down all tooth tissue. It acts on the inorganic a little more readily than on the organic. It does not form with tooth material, as highly soluble compounds, as does the agent, or agents, in causing chemical abrasion. The destroyed tissue will, in part, remain within the cavity of decay, but most of it can be wiped away with lint, or washed away by a stream of tepid water. Not nearly so much of the decayed material can be thus removed from a cavity formed by the colorless, or brown variety of caries.

Now, for the present, assume that white decay is immediately caused by nitric acid, and that this acid is formed by the oxidation of ammonia. As ammonia is composed of nitrogen and hydrogen, its oxidation results in nitric acid and water, Liebig and other authorities state that it is always thus oxidized in the presence of free oxygen. And if it was in Liebig's day, it is now. Remember, too, that ammonia always results from the putrefaction of nitrogenous organic compounds. Suppose an atom of nitric acid has got through the enamel—not through the hole that Mayr's bug goes in at—but through a defective spot. It acts on the dentine—faster on the lime than on the gelatin. A thin layer of the lime salts is dissolved and of course a thin layer of organic matter is left. By putrefaction it gives ammonia, to be oxidized into nitric acid, to dissolve more lime and expose more organic matter, to putrefy and give more ammonia, to be oxidized into more nitric acid, to dissolve more limesalts,—and thus, on and on, till the pulp cavity is reached. Such is white decay.

Now, is the presence of germs necessary to explain the burrowing found in this variety of caries, as is claimed by the germ theorists? If Dr. Sewill is right, that leptothrix are found only on the surface, they cannot burrow, as can nascent nitric acid manufactured on the spot, and getting into its work as fast as it is generated.

We might illustrate the most common, and the black decays in a similar way, but time, space and strength rebel.

A President of the Theological Seminary was asked by a somewhat alarmed pupil if he thought that Christianity could stand up against the present teachings of science. The calm old President asked: "What are the present teachings of science? I have not read the dailes this morning." It would be well for the germ theorists to agree on some general principle before asking us to abandon attainments made by years of research, and the most laborious and carefully conducted experiments. I have already alluded to the great liability to mistakes when using high powers, and that this liability increases in the ratio of the squares of the powers. Some of you remember the bitter disputes at the meeting of the American Dental Association, in Boston, in 1866, if my memory is corrcct, the basis of the disputation being, not principles nor deductions from observations made, but a simple disagreement as to what was actually then seen through the microscope.

And now we have a fresh illustration that these modern microscopic researches are to be taken with a good degree of allowance. I refer to the so-called discovery of Koch, who claims to have found out that tubercle is caused by a living parasite—the bacillus. Vast numbers claiming to be scientific, jumped with delight, showing a credulity worthy of marines and gossips, when this announcement was made. But now comes the *Chicago Medical Journal*, with the statement that it will soon publish an article by Dr. H. D. Schmidt, a distinguished microscopist of New Orleans, who claims that this bacillus is not an organized body, but a fat crystal. Dr. Schmidt declares he can produce, artificially, every form of Koch's bacillus. So again we see that chemistry is no exterminated by micro-zoology; but the point is the uncertainty of these high-power observations.

This paper is far too long; but I have felt it advisable to aim at covering the whole ground of disputation now, fearing I might never be able to resume and finish up, if a part were postponed. And though mainly written during a period of extreme physical suffering, I hope it will be read with profit.—*Ohio State Journal of Dental Science.*

